# HabiSign: a novel approach for comparison of metagenomes and rapid identification of habitat-specific sequences

**DOI:** 10.1186/1471-2105-12-S13-S9

**Published:** 2011-11-30

**Authors:** Tarini Shankar Ghosh, Monzoorul Haque Mohammed, Hannah Rajasingh, Sudha Chadaram, Sharmila S Mande

**Affiliations:** 1 Bio-sciences R&D Division, TCS Innovation Labs, Tata Consultancy Services Limited, 1 Software Units Layout, Madhapur, Hyderabad – 500081, Andhra Pradesh, India

## Abstract

**Background:**

One of the primary goals of comparative metagenomic projects is to study the differences in the microbial communities residing in diverse environments. Besides providing valuable insights into the inherent structure of the microbial populations, these studies have potential applications in several important areas of medical research like disease diagnostics, detection of pathogenic contamination and identification of hitherto unknown pathogens. Here we present a novel and rapid, alignment-free method called HabiSign, which utilizes patterns of tetra-nucleotide usage in microbial genomes to bring out the differences in the composition of both diverse and related microbial communities.

**Results:**

Validation results show that the metagenomic signatures obtained using the HabiSign method are able to accurately cluster metagenomes at biome, phenotypic and species levels, as compared to an average tetranucleotide frequency based approach and the recently published dinucleotide relative abundance based approach. More importantly, the method is able to identify subsets of sequences that are specific to a particular habitat. Apart from this, being alignment-free, the method can rapidly compare and group multiple metagenomic data sets in a short span of time.

**Conclusions:**

The proposed method is expected to have immense applicability in diverse areas of metagenomic research ranging from disease diagnostics and pathogen detection to bio-prospecting. A web-server for the HabiSign algorithm is available at http://metagenomics.atc.tcs.com/HabiSign/.

## Background

The advent of high-throughput sequencing technologies (and the concomitant emergence of the field of metagenomics) has facilitated the rapid sequencing and characterization of the entire genomic content obtained from various microbes present in a given environment [1]. By facilitating the recovery and characterization of genomic material from hitherto unculturable (and consequently unknown) microbes, the metagenomic approach enables researchers to gain valuable insights into the taxonomic and functional aspects of various microbial communities.

One of the primary goals of metagenomics projects is to perform a comparative analysis of microbial communities residing in diverse ecological niches. Assaying such differences can not only yield valuable insights into the inherent structure of these microbial communities, but can also identify genes/proteins/organisms that may confer specific functional characteristics to a given environment. Insights gained from such comparative studies are expected to have immense potential in several important areas of biological research, ranging from healthcare (e.g, disease diagnostics, detection of pathogenic contamination and characterization of novel pathogens), industrial biotechnology (bio-prospecting) and bio-remediation studies.

In order to perform a comparative analysis, the metagenomic samples need to be first characterized in taxonomic terms. Currently two approaches are used for characterizing taxonomic diversity. In the first approach, the 16S rRNA genes of various microbes present in a given environmental sample are extracted and sequenced. Subsequently, computational methods are employed for mapping these sequenced fragments to different taxonomic groups. In the second approach, the entire genomic content obtained from a given environment is extracted, sequenced and taxonomically characterized using computational methods [2-6]. The differences between the microbial communities are finally inferred by comparing the obtained taxonomic profiles.

Comparing metagenomic samples using the above approaches has the following limitations. First, a majority of sequences in metagenomic data sets originate from hitherto unknown organisms. Given that existing taxonomic characterization approaches are based on mapping metagenomic sequences to known taxonomic groups, adopting such approaches will result in a majority of sequences remaining un-characterized. The obtained taxonomic profiles are thus far from complete. Secondly, metagenomic data sets are huge and typically consist of millions of sequences. For example, the GOS data sets [7-9] contain more than 7 million sequences. Enormous time and/or computational resources is thus needed for characterizing all sequences in such huge data sets.

To address the above limitations, several recent studies have explored the utility of using differences in oligonucleotide usage patterns as a measure for comparing metagenomic data sets [10-12]. The reasons for using oligonucleotide usage patterns as features for comparing metagenomes are as follows. Previous studies have indicated that genomes of both prokaryotic and eukaryotic organisms possess distinct patterns of oligonucleotide usage (referred to as the 'genomic signature'), with closely related species having more similar patterns than distantly related ones [12-15]. For instance, correlations in tetranucleotide usage frequencies were used by methods such as TETRA for clustering individual genomic/metagenomic fragments [15]. Furthermore, recent studies have also indicated that environmental factors play a key role in determining the genomic signatures of the constituent organisms [10,11]. These studies have thus extended the concept of 'genomic signatures' to 'metagenomic signatures' [11]. Such 'signatures' obtained solely by profiling the overall oligonucleotide usage patterns of all sequences in metagenomic data sets, indirectly reflect the taxonomic composition of the underlying microbial communities. A distinct advantage of using the metagenomic signature approach is the following. Metagenomic signatures are obtained by profiling the compositional properties of all sequences in a given metagenomic data set irrespective of whether a given sequence is assigned to a known taxonomic group or not. The metagenomic signature is thus expected to comprehensively represent a given metagenome. Consequently, comparison of metagenomic signatures is expected to efficiently identify the differences between a set of metagenomes. An additional advantage of approaches based on comparison of oligonucleotide usage patterns is that they are much faster than alignment-based methods which involve an extensive process of aligning millions of sequences across metagenomic data sets.

In this paper, we present HabiSign – an approach that uses a novel methodology for generating the metagenomic signature. The performance of HabiSign has been tested on several metagenomic data sets having variations at phenotypic, species and biome levels. We demonstrate that HabiSign is able to group and cluster metagenomes much more efficiently than the average tetranucleotide frequencies based approach as well as the di-nucleotide relative abundance based approach used by Willner *et al.*[11]. In addition, we also demonstrate that the signature generated using the HabiSign approach aids in identifying subsets of sequences that are specific to given metagenomic samples. Identifying the taxonomic affiliation of such subsets of sequences (and the genes/proteins encompassed within them) may help in determining the key players which are possibly responsible for conferring a specific phenotype to a given metagenome.

## Results

### Principle of HabiSign algorithm

The principle of the HabiSign algorithm is as follows. Given that environmental factors influence the overall oligonucleotide composition of the genomes of the constituent organisms [10,11], the oligonucleotide usage patterns of the DNA sequences collected from diverse habitats are expected to be different. Consequently, if a 'feature vector space' is created, wherein the DNA sequences are represented as distinct points (on the basis of their oligonucleotide usage patterns), sequences belonging to a particular metagenome are likely to localize to specific regions in this space. This pattern of spatial localization will essentially capture the signature corresponding to a given metagenome. Furthermore, this pattern of localization is expected to be different for sequences originating from metagenomes sampled from diverse habitats. Consequently, quantifying these differences can primarily aid in efficiently comparing and grouping metagenomes. Furthermore, while comparing a set of metagenomes obtained from diverse habitats, it is possible to identify regions (in the same feature space) that are selectively over-mapped by sequences belonging to metagenome(s) sampled from a particular habitat. The sequences mapping to such regions are likely to originate from organism(s) or gene cluster(s) that are specific to a particular habitat.

### HabiSign algorithm

#### Identification of reference points

In order to study the pattern of localization of sequences constituting metagenomic data sets, it is necessary to first identify a set of reference points in the feature vector space. It is desirable that these reference points are not only spatially well separated, but also represent diverse patterns of oligonucleotide compositions generally observed across biological sequences. The mapping pattern of the sequences (constituting a given metagenome) to these reference points will thus serve as the 'metagenomic signature'.

For the identification of these reference points, the following procedure was adopted. Sequences corresponding to 237 completely sequenced microbial genomes (one representative from each genus) were downloaded from the NCBI database (ftp://ftp.ncbi.nih.gov/genomes/Bacteria/all.fna.tar.gz). The rationale behind selecting 237 genomes is the following. Selecting genomes from each of the 237 genera ensures that the generated reference points comprehensively represent diverse patterns of oligonucleotide usage generally observed across known biological realm. In addition, selecting one representative from each genera ensures that the generated reference points are not too many, and therefore does not adversely impact the the overall computation time.

Each genome was split into non-overlapping fragments of 1000 base pair (bp) length. For each fragment, a 128 dimensional vector containing the frequencies of all possible 256 tetra-nucleotides (with frequencies of complementary tetranucleotides, e.g, ACGT and TGCA, being counted together) was computed and stored. Reducing the dimensionality of the vector from 256 to 128 was done to reduce the overall computation time. Subsequently, using Manhattan distance (between individual vectors) as the similarity criterion, these vectors were then clustered by adopting the standard k-means clustering approach [16]. A critical aspect of the k-means clustering approach is deciding on the number of clusters (k) to be generated for the sequence fragments. Previous studies [17] have suggested a simple thumb rule wherein, the number of clusters (k) is given by:

where n is the number of observations . In this case, n is equal to the total number of sequence fragments generated from the 237 microbial genomes.

Using the above rule, a total of 631 clusters were created. Vectors corresponding to the centroid of each individual cluster were computed and stored along with the total number of sequences in the respective cluster. These 631 centroid vectors were considered as distinct 'reference points' (hereafter referred to as RPs) and represented the different regions of feature vector space. It is to be noted that clustering step described above is a one-time activity.

#### Generation of metagenomic signature

For each sequence in a given metagenome, the frequencies of all possible tetra-nucleotides is calculated and represented in the form of a 128 dimensional vector (as described in the previous section). The distance of this vector to each of the 631 RPs is computed. The closest RP (in terms of distance) as well those RPs having a distance less than or equal to 1.01 times the distance of the closest RP are identified. The identified set of RPs is referred to as 'hit profile' for that particular sequence. This hit profile indirectly represents the composition of the given sequence as well as the spatial localization of this sequence in feature vector space. Hit profiles for all sequences constituting a metagenome are obtained in a similar manner. The propensity (H_ij_) of a RP_i_ to be mapped by sequences belonging to a given metagenome j is then calculated using the following formula:

Where,

C_ij_ is the number of times i^th^ RP (i.e. RP_i_) is picked up by the hit profile of sequences in metagenome j, N_j_ is the total number of sequences in metagenome j. F_i_ denotes the frequency of genomic fragments mapping to the cluster corresponding to the given RP_i_ in the k-means clustering process.

F_i_ is calculated using the following formula:

Given that metagenomes exhibit distinct oligonucleotide usage patterns, the propensity with which each of the 631 RPs are mapped are expected to be different. The propensity of all the 631 RPs for a given metagenome are then represented as a 631-dimensional vector of the form [*H*_1_, *H*_2_…*H_i_*,…*H*_631_]. This vector (henceforth referred to as 'HabiSign signature') thus indicates the pattern of localization of the sequences present in a given metagenome to spatially distinct regions in feature vector space.

#### Identification of degree of relatedness across metagenomes

For a given pair of metagenomic data sets (j and k), the Manhattan distance (L1 norm) between their HabiSign signatures is first computed using the following formula:

where, H_ij_ and H_ik_ represent the propensity of a RP_i_ to be mapped by sequences belonging to metagenomes j and k respectively.

To compare a set of m metagenomes, the pairwise distance values are used for generating a m x m distance matrix. Elements of this matrix indicate the degree of relatedness across the different metagenomes in the set. Relatedness between metagenomes can also be visualized by providing this distance matrix as an input to a suitable tree-building software package such as Phylip [18].

### Identification of habitat-specific sequences

Metagenomes obtained from diverse habitats exhibit different patterns of oligonucleotide usage. Consequently, the propensity with which RPs are mapped (by sequences present in the metagenomes) is expected to be different. By identifying RPs over-mapped by sequences belonging to a specific habitat (as compared to the other), it is possible to identify sequences that are specific to a given habitat. The procedure adopted for identifying such sequences is as follows. Given two metagenomes corresponding to two different habitats (j and k), the relative mapping propensity (for each RP) is first calculated as the ratio of the mapping propensity obtained with habitat j (H_ij_) to that obtained with habitat k (H_ik_). While RPs having a relative mapping propensity with z score value greater than 2.5 are identified as specific to habitat j, those RPs having a relative mapping propensity with z score value lower than -2.5 are identified as specific to habitat k. The identified sets of habitat specific RPs are further refined using a Bayesian probabilistic approach, wherein only those specific RPs are retained which show a strong co-occurring tendency within the hit profiles obtained for a particular habitat. Sequences of a metagenome that map to the corresponding refined set of RPs (identified as specific for this metagenome) are tagged as specific to that metagenome. To further improve the confidence on the identified specific sequences, the procedure described above is repeated multiple times using a different set of reference points. A different set of reference points can be generated using the same procedure described earlier (in section 'Identification of reference points') with a different set of genomes.

## Validation

Besides being able to capture differences in oligonucleotide usage patterns between metagenomes sampled from diverse habitats, an ideal metagenomic signature should also be able to identify subtle differences (at organismal or phenotypic level) between metagenomes sampled from similar habitats. Consequently, validation of HabiSign was performed using the following three sets of metagenomes (Additional File 1).

### (A) Metagenomes from diverse habitats

Metagenomes from diverse habitats, including those sampled from freshwater, marine, coral reefs, hot springs, salterns, etc, were downloaded from http://www.theseed.org/DinsdaleSupplementalMaterial[19]. The hot spring metagenomic data sets were obtained from NCBI (http://www.ncbi.nlm.nih.gov/) .

### (B) Metagenomes from similar habitats but sampled from diverse species

Gut metagenomes sampled from cow, chicken, mice, human and fish were downloaded from http://www.theseed.org/DinsdaleSupplementalMaterial[19].

### (C) Metagenomes from similar habitats and species but exhibiting different phenotypes

Sequences belonging to five mouse gut metagenomes (previously analyzed by Turnbaugh *et al.*[20]) were downloaded from http://www.ddbj.nig.ac.jp. While three of these metagenomes (Lean1, Lean2 and Lean3) were obtained from the gut of lean mice, two of them (Obese1 and Obese2) were obtained from the gut of obese mice. These data sets thus represented metagenomes obtained from similar habitats in similar species but nevertheless corresponded to two functional phenotypes namely lean and obese respectively.

### Comparison of the performance of HabiSign with other approaches

The clustering patterns obtained with the HabiSign signatures (for the three sets of metagenomes described above) were also compared with those obtained using two metagenomic signature approaches described below:

### (A) Average Tetra-nucleotide based approach

In the average tetra-nucleotide nucleotide frequency based approach, the signature (for a given metagenome) is obtained by simply computing the average tetranucleotide frequencies observed across all sequences belonging to the metagenome (as a 128 dimensional vector as described in the section 'Identification of reference points'). The relative distances between any two metagenomes (J and K) are then computed as a simple Manhattan distance between the two vectors.

### (B) Dinucleotide relative abundance based approach

The dinucleotide relative abundance based approach was adopted by Willner et al. [11] for comparing/clustering metagenomic data sets. In this approach, the propensity of each dinucleotide for over- or under-representation in a metagenome is first obtained and represented in the form of a 16 dimensional vector. This vector represents the signature for a given metagenome. The above procedure (described in Willner et al. [11]) was implemented in Perl. This implementation was used for generating the signatures for the metagenomic data sets used in the present study.

The clustering pattern obtained for the three methods (graphically represented in the form of trees) were first qualitatively compared. Subsequently, genealogical sorting indexes [21] were computed for obtaining a precise quantitative measure of the clustering efficiencies of each of the methods. Given a set of observations belonging to different groups (represented in the form of a hierarchical tree), the Genealogical Sorting Index (GSI) value obtained for a particular group provides a quantitative measure of how closely the observations belonging to this group have clustered in the given tree. The GSI value ranges between the values of 0 and 1, wherein a group obtains a GSI value of 1, if all the members of the group can be distinctly represented as a separate homogenous sub-clade of the tree (having no members belonging to any of the other groups). As the observations (belonging to a particular group) spread out, the GSI values for such groups fall below one. Given these attributes, GSI values can be used as a quantitative measure of the resolving power of different methods to distinguish between metagenomes belonging to different groups. A detailed description of the computation of genealogical sorting indexes and their comparison for the three different methods is given in Additional File 2.

### Validation results

#### Analyzing signatures of metagenomes sampled from diverse habitats

**Figure 1:**
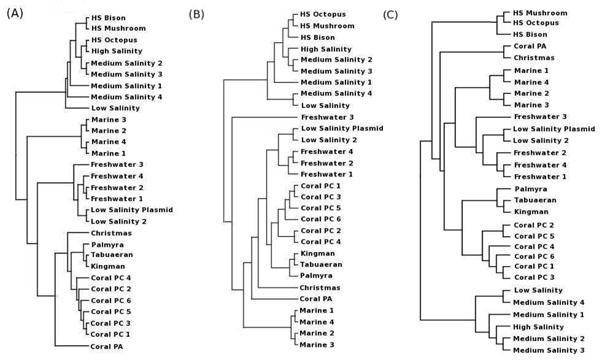
**Clustering pattern of metagenomic signatures corresponding to the diverse aquatic metagenomes**. Clustering patterns obtained using (A) HabiSign signatures and (B) metagenomic signatures generated using the average tetranucleotide frequency approach ( C) Dinucleotide relative abundance based approach.

A distance matrix was generated by comparing HabiSign signatures corresponding to metagenomic data sets sampled from diverse aquatic habitats. These signatures were then hierarchically clustered by providing this distance matrix as an input to the neighboring joining program of PHYLIP software package [18] (neighbor joining algorithm with default parameters, branch lengths not specified). Figure 1A graphically illustrates the results of this analysis.

Results (Figure 1A) indicate a clustering pattern, wherein the HabiSign signatures corresponding to metagenomes obtained from similar habitats are observed to cluster together. For instance, HabiSign signatures of metagenomes obtained from the saline habitats are observed to cluster together, and are distinctly separated from those belonging to the metagenomes sampled from freshwater habitats. Similarly, it is also observed that the HabiSign signatures of the four metagenomic samples taken from the coral atolls in the Northern Line islands (Christmas, Palmyra, Tabuaeran, and Kingman) cluster together along with those of the seven coral reef-associated samples (Coral PC 1-6, Coral PA). However, the HabiSign signature of the coral metagenome from Porites astreoides (Coral PA) was observed to be an outlier with respect to the cluster containing the HabiSign signatures corresponding to the other coral reef metagenomes (Coral PC 1-6). A similar pattern of clustering was also obtained earlier by Willner *et al.*, [11] who suggested two likely reasons for the Coral PA sample being observed as an outlier. First, these anomalies could be due to artifacts accumulated during the sample preparation stage. The second reason could be the presence of specific taxonomic groups (in the Coral PA sample) that possess a distinct oligonucleotide composition as compared to the other coral reef samples. In order to check the latter possibility, taxonomic profiles of all the coral reef associated samples were obtained using SPHINX [5]. A comparison of these taxonomic profiles (Additional File 3) indicates that the Coral PA sample has a distinct taxonomic composition as compared to coral reef-associated samples (Coral PC 1-6). For instance, the Coral PA sample is observed to be noticeably depleted in its Cyanobacterial, Firmicutes and Spirochaetes content as compared to the other six coral reef associated samples. Given these observations, it is likely that the distinct oligonucleotide composition observed for the Coral PA sample (reflected in its HabiSign signature) is due to the specific differences in the abundance patterns of the above mentioned taxonomic groups. Moreover, the taxonomic composition of the Coral PA sample is observed to be similar to that of the sample isolated from the Christmas coral atoll.

To further analyze the above aspects, we used HabiSign to identify sequences ‘specific’ to the Coral PA habitat and the Coral PC habitats. These sets of ‘habitat specific’ sequences have oligonucleotide usage patterns specifically over-represented in Coral PA and Coral PC habitats respectively. Taxonomic analysis of these sequences is expected to provide a direct indication of specific taxonomic groups that differentiate the two habitats. A comparison of taxonomic assignments (at phylum level) of sequences identified as specific to the Coral PA and Coral PC 1-6 metagenomes respectively is provided as a table in Additional File 4. Results in this table indicate that the taxonomic assignment patterns obtained with specific sequences are similar to those obtained using the entire Coral PA and Coral PC 1-6 metagenomes. As observed earlier (Additional File 3), sequence dataset identified as specific to the Coral PA habitat is enriched for the phyla Proteobacteria, Euryarchaeota, Thermotogae and Planctomycetes. Besides, there is a marked depletion in the proportion of Cyanobacteria, Firmicutes and Actinobacteria. Thus, the similarities/differences in taxonomic profiles are clearly reflected in clustering of the respective HabiSign signatures (Figure 1A).

Furthermore, Figure 1A indicates a clustering pattern wherein HabiSign signatures corresponding to the saline metagenomic samples have been progressively arranged as per their salinity levels. The only exception to this pattern are the HabiSign signatures corresponding to the two low salinity samples, namely Low salinity 2 and Low salinity Plasmid. The HabiSign signatures of the latter two are seen to be clustered along with those corresponding to the metagenomic samples obtained from freshwater habitats. To check the possible reason for this clustering pattern, taxonomic profiles of these two samples were compared with the taxonomic profiles of other samples obtained from saline and freshwater habitats. Results of this analysis (Additional File 5) indicate an over abundance of Firmicutes (and depletion of Actinobacteria) in the four freshwater samples, the Low Salinity 2 and Low salinity Plasmid samples as compared to samples obtained from other saline habitats. This clearly indicates the ability of the HabiSign signature in suggesting taxonomic differences present in different metagenomes.

In contrast to the clustering pattern observed for the HabiSign signatures (Figure 1A), it is observed that the average tetranucleotide frequency approach incorrectly places the metagenomic signatures of the three Freshwater metagenomes (Freshwater 1,2 and 4) close to the coral and the marine metagenomes and farther from the Freshwater 3 metagenome (Figure 1B). A similar picture is also obtained using the dinucleotide relative abundance approach, wherein, the signatures of Low Salinity Plasmid and Low Salinity metagenomes are placed closer to the Freshwater metagenomes (Freshwater 1,2 and 4) as compared to the Freshwater 3 metagenome (Figure 1C). Furthermore, it is also observed that metagenomic signatures generated using the average tetranucleotide frequency approach for the coral samples (PC 1-6) are placed away from the other coral reef associated sample (Coral PA) and are seen to be placed closer to those obtained for the Freshwater and the Low salinity metagenomes. It is also observed that using the dinucleotide relative abundance approach [11], places the signature of the Coral PA sample even farther away from the Coral PC samples and relatively closer to the Marine, Freshwater and Low salinity samples (Figure 1C). These results (Figure 1) thereby indicate that, unlike the HabiSign approach, the average tetranucleotide frequency approach as well as the dinucleotide relative abundance based approach are unable to accurately distinguish between the oligonucleotide usage patterns obtained from the coral, marine, low salinity and the freshwater habitats. On the other hand, the HabiSign approach is able to clearly distinguish between the oligonucleotide usage patterns for the above habitats and is able to place the generated signatures into distinct clades in the hierarchical tree.

The high clustering efficiency of HabiSign approach is further evident in the comparative analysis of GSI values (Additional File 2) obtained for the different biome level groups corresponding to the aquatic metagenomes. For most of the biome level groups (with exception of the Hot Spring biome), it is observed that the GSI values obtained using HabiSign are higher than or equal to those obtained using the average tetranucleotide frequency or the dinucleotide relative abundance approaches (Additional File 2).

#### Analyzing signatures of metagenomes from similar habitats but sampled from diverse species

**Figure 2:**
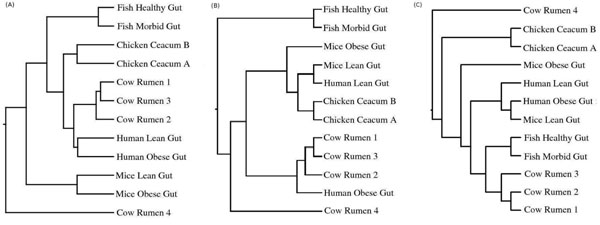
**Clustering pattern of metagenomic signatures corresponding to the gut metagenomes from diverse species**. Clustering patterns obtained using (A) HabiSign signatures and (B) metagenomic signatures generated using the average tetranucleotide frequency approach ( C) Dinucleotide relative abundance based approach

The signatures for the gut metagenomes of diverse species (namely, Cow, Fish, Chicken, Human and Mice), obtained using the HabiSign approach as well as the average tetranucleotide frequency approach, were hierarchically clustered as described in the previous section. Figure 2 graphically illustrates the results of this analysis. Similar to the results obtained with metagenomes from diverse habitats, HabiSign signatures corresponding to gut samples obtained from diverse species show a nice clustering pattern (Figure 2A). HabiSign signatures obtained from the gut samples of the same species are seen to be clustered together indicating similar oligonucleotide usage patterns within microbial communities residing in a given species. Cow Rumen sample 4 is observed to be the only exception to the above pattern (Figure 2A). The taxonomic compositions of the cow rumen samples indicated a significant overabundance of Proteobacteria as well as a depletion of Firmicutes, Bacteroidetes and Euryarchaeota in the Cow rumen sample 4 (Additional File 6). Thus, the outlier observed in the tree generated using HabiSign signatures suggests differential presence of certain microbial groups.

Similar to the analysis performed for the Coral Samples, HabiSign was used to identify sequences ‘specific’ to the Cow Rumen 4 and the Cow Rumen 1-3 metagenomes. Analyzing the phylum level taxonomic affiliations of these ‘specific’ sequences (Additional File 7) reveals a picture similar to that obtained by the analyzing the overall phylum compositions of the corresponding metagenomes (Additional File 6). Interestingly, the phylum Firmicutes which was observed to be under-represented in overall phylum composition of the Cow Rumen 4 metagenome was observed to be over-represented in the sequences identified as specific to the Cow Rumen 4 (as compared to the other Cow Rumen metagenomes). A probable reason for this could be the presence of a specific hitherto unknown species of Firmicutes (having unique oligonucleotide composition) in the Cow Rumen 4 sample.

A comparison of the clustering pattern obtained using HabiSign signature (Figure 2A) with that obtained using the average tetranucleotide frequency approach (Figure 2B) and the dinucleotide relative abundance based approach (Figure 2C) indicates the following. Similar to the clustering pattern obtained using HabiSign signatures, the metagenomic signature of the Cow Rumen 4 sample (generated using both the approaches) is observed to be an outlier to those corresponding to the other cow rumen samples. However, it is also observed in Figure 2B that the metagenomic signature of the Mice Lean Gut (generated using the average tetranucleotide frequency approach) is placed closer to the signatures corresponding to the Human Lean Gut and the Chicken Caecum samples than to the other mice gut sample, namely Mice Obese Gut. A similar pattern of clustering is also obtained with the dinucleotide relative abundance based approach where in, the mouse lean gut is placed closer to the Human Gut, Fish gut and the Cow Rumen 1-3 samples as compared to the Mouse Obese gut sample. This is in contrast to the clustering pattern using the HabiSign approach, where in the HabiSign signatures obtained for both the mouse gut samples are observed to be placed together in a distinct clade (Figure 2A). These differences in the clustering pattern are also reflected in the GSI values obtained for the Mouse samples (Additional File 2). While HabiSign generates a GSI value of 1 for the mouse samples, the corresponding GSI values (for the mouse samples) obtained using average tetranucleotide frequency based approach and the dinucleotide relative abundance based approach are relatively much lower (0.27 and 0.18 respectively). Similarly, it is also observed that (in contrast to the HabiSign signatures), the metagenomic signatures (generated using the average tetranucleotide frequency approach) for the two human gut samples are placed farther apart in hierarchical tree (Figure 2B). Similarly, it is observed in Figure 2C that using the dinucleotide relative abundance based approach, the metagenomic signature of the Human Obese Gut generated is placed closer to the Mice Lean Gut as compared to that corresponding to the other Human Gut sample. These discrepancies in clustering pattern indicate that, unlike the HabiSign approach, the metagenomic signatures generated using the average tetranucleotide frequency approach, are not able to efficiently distinguish between the species specific variations in oligonucleotide usage patterns. These differences in the clustering efficiencies (for the human samples) are also reflected in the corresponding GSI values obtained using the three methods on the human samples (Additional File 2). While HabiSign generates a GSI value of 1 for the human samples, the corresponding GSI values obtained using average tetranucleotide frequency based approach and the dinucleotide relative abundance based approach are 0.12 and 0.45 respectively.

#### Analyzing signatures of metagenomes identical at habitat and species level but exhibiting differences at phenotypic level

**Figure 3:**
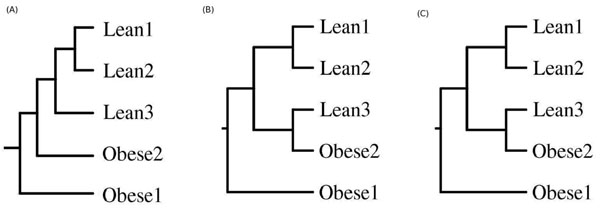
**Clustering pattern of metagenomic signatures corresponding to the gut metagenomes from lean and obese mice**. Clustering patterns obtained using (A) HabiSign signatures and (B) metagenomic signatures generated using the average tetranucleotide frequency approach ( C) Dinucleotide relative abundance based approach

Figure 3 illustrates the pattern of clustering obtained with the signatures corresponding to the five mouse gut metagenomes, using the HabiSign approach (Figure 3A) and the average tetranucleotide frequency approach (Figure 3B). It is observed that the HabiSign signatures corresponding to the lean mouse samples cluster separately from those of obese mouse samples (Figure 3A). These results demonstrate that HabiSign signature is able to efficiently discriminate between microbial communities having subtle differences at the phenotypic level. It is also observed that, amongst the three lean samples, the Lean 3 sample is the closest to the two obese samples. Similarly, it is observed in Figures 3B and 3C that the signature of the Lean 3 sample, obtained using the average tetranucleotide frequency approach as well as the dinucleotide relative abundance based approach, in placed closer to the Obese 2 sample than to the other lean samples. These differences in the clustering efficiencies (for the mouse samples) are also reflected in the corresponding GSI values obtained using the three methods on these samples (Additional File 2).

In order to find a reason for the general proximity of the lean 3 sample to the obese samples, an analysis of the five mouse gut metagenomes were performed using SPHINX [5]. These results (Additional File 8) revealed similarities in the taxonomic composition of the Lean3 sample and the obese samples. The Bacteroidetes/Firmicutes ratio of the Lean3 sample (3.51) was observed to be more similar to the Obese1 and Obese2 samples (2.46 and 3.40 respectively). In contrast, the average Bacteroidetes/Firmicutes ratio of the lean samples was 8.17. The above result is also reflected in Figure 3A, wherein, the Lean1 and Lean2 samples (with similar taxonomic composition) are observed to have clustered closer to each other than to the Lean3 sample (having a deviant taxonomic profile). These results further demonstrate the robustness of the signatures generated using the HabiSign approach.

To further demonstrate the utility of the HabiSign approach in identifying sequences specific to a given habitat, sequences specific to lean and obese mouse guts were identified adopting the methodology described in the section 'Identification of habitat-specific sequences'. The results of this analysis (Table 1) indicate a 2.4 to 4.7 fold increase in the Bacteroidetes/Firmicutes ratio in the lean specific sequences identified by HabiSign. Similarly, the obese specific sequences were observed to be significantly enriched for sequences originating from Firmicutes, reflected as 3.6 – 6.1 fold decrease in the Bacteroidetes/Firmicutes ratio (Table 1). An earlier study on these metagenomic data sets had indicated a higher Bacteroidetes/Firmicutes ratio in the gut metagenomes of lean individuals as compared to those from obese individuals [19]. Given this observation (also reflected in the taxonomic profiles obtained using SPHINX), sequences identified (by HabiSign) as specific to lean samples are expected to have a relatively higher Bacteroidetes/Firmicutes ratio. Similarly the obese phenotype-specific sequences (identified by HabiSign) are expected to be significantly enriched for Firmicutes, thereby resulting in a relatively lower Bacteroidetes/Firmicutes ratio. The results obtained in this study reaffirm that the HabiSign signature not only helps in comparing and grouping metagenomes, but also aids in identifying a subset of sequences that are unique to a metagenomic sample(s).

**Table 1:** Analysis of lean and obese specific sequences

Sample	Bacteroidetes/Firmicutes Ratio		Fold increase in Bacteroidetes/Firmicutes ratio
		
	All lean sequences	Lean-specific sequences	
Lean 1	7.82	24.1	3.08

Lean 2	8.52	20.2	2.37

Lean 3	3.51	14.89	4.24



**Sample**	**Bacteroidetes/Firmicutes Ratio**		**Fold decrease in Bacteroidetes/Firmicutes ratio**
		
	**All obese sequences**	**Obese-specific sequences**	

Obese 1	2.47	0.69	3.58

Obese 2	3.4	0.56	6.07

A comparison of Bacteroidetes/Firmicutes ratio observed in lean and obese mouse gut metagenomes as well as in the sequence data sets identified (by HabiSign) as lean specific and obese specific.

## Discussion

Genomes of microbes inhabiting a particular habitat display distinct patterns of oligonucleotide usage. The present study utilizes these habitat-specific patterns for generating a 'metagenomic signature'. The approach used for generating the signatures is distinct from those used by earlier studies by Willner *et al.*[11]. In the latter study, the metagenomic signature was computed by averaging the frequencies of all possible di-nucleotides present in sequences constituting a given metagenome. The signature generated using such an approach is able to capture the differences in the overall oligonucleotide usage patterns observed across metagenomes. However, the applicability of such a signature is limited for the following reason. Consider a set of metagenomes sampled from highly similar habitats. In such cases, the overall oligonucleotide usage patterns of a majority of species constituting these metagenomes are expected to be more or less similar. However, subtle differences (in nucleotide usage) may exist with respect to a small subset of species (or a gene cluster) constituting these metagenomes. A metagenomic signature computed by averaging the oligonucleotide frequencies of all sequences (in a given metagenome) may primarily fail to efficiently capture such subtle differences. This is also evident in our analysis of real metagenomes, where it is observed that both the average tetranucleotide frequency approach as well as the dinucleotide relative abundance based approach (as used by Willner et. al. [11]) fail to efficiently capture these differences in oligonucleotide usage patterns. To further validate these observations, a comparative analysis of the clustering efficiencies of various methods was performed using simulated metagenomic data sets. The results (see Additional File 9) of this analysis (using GSI as a measure) clearly indicate that, unlike HabiSign, the performance of 'generic' signature approaches drop when the analyzed metagenomic data sets have subtle differences in their taxonomic composition. Moreover, the utility of such 'generic' signatures is limited since they cannot be used for identifying habitat-specific sequences. In contrast, the HabiSign signature first partitions sequences constituting a given metagenomic data set into spatially distinct groups, wherein all sequences in each group possess a distinct pattern of oligo-nucleotide usage. In other words, the vector corresponding to the HabiSign signature (for a given metagenome) efficiently captures the variations (however subtle) in a given metagenome (instead of simply averaging them out). Comparing such vectors obtained from two sets of metagenomes can thus be used to identify groups (however small) that are distinct to either metagenomes. Sequences mapping to these groups can be identified as specific to the respective metagenome(s). Organisms and the genes corresponding to or encompassed by these specific sequences can potentially be studied to identify factors conferring a specific phenotype that provide an adaptive advantage to a given microbial community. Identification of such factors may have immense potential applications in several areas ranging from disease diagnostics to bio-prospecting. Interestingly, the HabiSign approach is alignment-free and involves simple numerical calculations. Consequently, this approach is extremely rapid as compared to existing comparative metagenomic approaches which rely on generating alignments between millions of sequences constituting metagenomic data sets.

In contrast to the k-mer size of 2 used by Willner *et al*. [11] for generating the metagenomic signature, a k-mer size of 4 was used in the present study for the following reason. Earlier studies have indicated the suitability of tetra-nucleotide frequencies for taxonomic discrimination [15,22]. In addition, the lengths of sequences in metagenomic data sets generated using existing sequencing technologies generally have lengths in the range of 100 to 1000 bp. For sequences in this length range, using lower k-mer sizes (< 4) results in lower discriminatory power. In contrast, oligonucleotide frequency values obtained with higher k-mer sizes (greater than or equal to 5) are expected to be significantly low and statistically insignificant.

Given that both HabiSign as well as other approaches (used in the present study) involve simple vector based mathematical calculations, the difference in the overall execution time of the methods is marginal (with HabiSign being 2-3 minutes slower per million sequences). Although HabiSign is slower (marginally), it has the added advantage of identifying habitat-specific sequences. Moreover, overall results of the validation studies presented in this paper indicate that the metagenomic signatures obtained using HabiSign are much more robust as compared to other approaches.

## Conclusions

The present study describes a rapid and accurate approach for capturing habitat specific oligonucleotide usage patterns in the form of a metagenomic signature. Results with metagenomic data sets indicate that the present approach is successful in differentiating between metagenomes having variations at phenotypic, species and biome levels. Moreover, the present approach is also able to identify sequences that are 'specific' to a given habitat. Identification of such habitat specific sequences is expected to have immense utility in several areas of life sciences research.

## Authors' contributions

TSG, HR, MMH and SSM have conceived the idea and designed the detailed methodology. TSG, SC and HR have implemented the algorithm, created validation datasets and carried out detailed validation and testing of the algorithm. TSG, MMH, HR and SSM have analyzed the data and finally drafted the complete paper.

## Competing interests

The authors declare that they have no competing interests.

## Supplementary Material

Additional file 1 **Details of microbial metagenomes used in this study** A pdf document containing the details of the metagenomes used in the study. These details include the Name of the metagenome, the NCBI genome project id, the the biome corresponding to the metagenome and the associated reference. Click here for file

Additional File 2 **Computation and Comparison of Genealogical Indexes (GSIs)** A pdf document describing the details of the computation and comparison of GSI values. These values are obtained for the various biome-level, species-level and phenotypic groups using the three methods (namely, HabiSign, the average tetranucleotide frequency approach and the dinucleotide relative abundance based approach) Click here for file

Additional file 3 **Distribution of taxonomic assignments for the coral reef associated metagenomes** A pdf document containing the distribution of taxonomic assignments (cumulated at phylum level) obtained using SPHINX for the coral reef associated metagenomes. Click here for file

Additional file 4 **Taxonomic analysis of sequences identified as specific to the Coral PA and the Coral PC metagenomes** A pdf document containing the distribution of taxonomic assignments (cumulated at phylum level) obtained using SPHINX for the sequences identified as specific to the Coral PA and Coral PC (Coral PC 1-6) metagenomes. Click here for file

Additional file 5 **Distribution of taxonomic assignments for metagenomes obtained from habitats of varying salinity** A pdf document containing the distribution of taxonomic assignments (cumulated at the phylum level) obtained using SPHINX for the metagenomes sampled from habitats of varying salinity. Click here for file

Additional file 6 **Distribution of taxonomic assignments for the cow rumen metagenomes** A pdf document containing the distribution of taxonomic assignments (to various phyla) obtained using the SPHINX algorithm for the cow rumen metagenomes. Click here for file

Additional file 7 **Taxonomic analysis of sequences identified as specific to the Cow rumen 4 and Cow Rumen 1-3 metagenomes** A pdf document containing the distribution of taxonomic assignments (cumulated at phylum level) obtained using SPHINX for the sequences identified as specific to the Cow Rumen 4 and the other Cow Rumen (Cow Rumen 1-3) metagenomes. Click here for file

Additional file 8 **Distribution of taxonomic assignments from lean and obese mouse gut metagenomes**. A pdf document containing the distribution of taxonomic assignments (to various phyla) obtained using the SPHINX algorithm for the lean and obese mouse gut metagenomes. Click here for file

Additional file 9 **Genealogical Sorting Index (GSI) based comparative analysis of clustering efficiencies using simulated metagenomic data sets**. A pdf document describing a GSI based comparative analysis of the clustering efficiencies of HabiSign, the average tetranucleotide frequency approach and the dinucleotide relative abundance based approach on simulated metagenomic data sets Click here for file
