# Supplementary material for: HabiSign: a novel approach for comparison of metagenomes and rapid identification of habitat-specific sequences
Source: BMC Bioinformatics. 2011 Nov 30;12(Suppl 13):S9. doi: 10.1186/1471-2105-12-S13-S9 (PMC3278849; doi:10.1186/1471-2105-12-S13-S9)
Supplement: Additional file 1 — Details of microbial metagenomes used in this study A pdf document containing the details of the metagenomes used in the study. These details include the Name of the metagenome, the NCBI genome project id, the the biome corresponding to the metagenome and the associated reference. [file 1471-2105-12-S13-S9-S1.pdf]

Supplementary Table 1: Details of microbial metagenomes used in this study

Name	NCBI Genome project ID #	Biome	Reference
Acid mine	13696	Mine drainage	Tyson *et al.*, 2004
Lean mouse gut 1	17391	Animal associated	Turnbaugh *et al.*, 2006
Lean mouse gut 2	17393	Animal associated	Turnbaugh *et al.*, 2006
Lean mouse gut 3	17395	Animal associated	Turnbaugh *et al.*, 2006
Obese mouse gut 1	17397	Animal associated	Turnbaugh *et al.*, 2006
Obese mouse gut 2	17399	Animal associated	Turnbaugh *et al.*, 2006
Healthy fish gut	28389	Fish associated	Dinsdale *et al.*, 2008
Morbid fish gut	28391	Fish associated	Dinsdale *et al.*, 2008
Cow Rumen 1	28607	Animal associated	Dinsdale *et al.*, 2008
Cow Rumen 2	28605	Animal associated	Dinsdale *et al.*, 2008
Cow Rumen 3	28611	Animal associated	Dinsdale *et al.*, 2008
Cow Rumen 4	28609	Animal associated	Dinsdale *et al.*, 2008
Chicken ceacum A	28597	Animal associated	Dinsdale *et al.*, 2008
Chicken ceacum B	28599	Animal associated	Dinsdale *et al.*, 2008
Lean human gut	32089	Animal associated	Turnbaugh *et al.*, 2009
Obese human gut	32089	Animal associated	Turnbaugh *et al.*, 2009
Porites compressa 1	28433	Coral	Dinsdale *et al.*, 2008
Porites compressa 2	28431	Coral	Dinsdale *et al.*, 2008
Porites compressa 3	28429	Coral	Dinsdale *et al.*, 2008
Porites compressa 4	28435	Coral	Dinsdale *et al.*, 2008
Porites compressa 5	28427	Coral	Dinsdale *et al.*, 2008
Porites compressa 6	28437	Coral	Dinsdale *et al.*, 2008
Porites astreoides	28371	Coral	Willner *et al.* 2009
Coral reef: Kingman	28343	Marine	Dinsdale *et al.*, 2008 a
Coral reef: Palmyra	28363	Marine	Dinsdale *et al.*, 2008 a
Coral reef: Tabuaeran	28367	Marine	Dinsdale *et al.*, 2008 a
Coral reef: Christmas	28347	Marine	Dinsdale *et al.*, 2008 a
Marine 1	19145	Marine	Mou *et al.*, 2008
Marine 2	19145	Marine	Mou *et al.*, 2008
Marine 3	19145	Marine	Mou *et al.*, 2008
Marine 4	19145	Marine	Mou *et al.*, 2008
Freshwater 1	28407	Freshwater	Dinsdale *et al.*, 2008
Freshwater 2	28405	Freshwater	Dinsdale *et al.*, 2008

Freshwater 3	28603	Freshwater	Dinsdale *et al.*, 2008
Freshwater 4	28387	Freshwater	Dinsdale *et al.*, 2008
Low salinity 1	28359	Hypersaline	Dinsdale *et al.*, 2008
Low salinity 2	28461	Hypersaline	Dinsdale *et al.*, 2008
Low salinity plasmids	28443	Hypersaline	Dinsdale *et al.*, 2008
Medium salinity 1	28449	Hypersaline	Dinsdale *et al.*, 2008
Medium salinity 2	28459	Hypersaline	Dinsdale *et al.*, 2008
Medium salinity 3	28379	Hypersaline	Dinsdale *et al.*, 2008
Medium salinity 4	28377	Hypersaline	Dinsdale *et al.*, 2008
High salinity	28453	Hypersaline	Dinsdale *et al.*, 2008
HS Bison	34801	Thermal	Bhaya D *et al.*, 2007
HS Octopus	20725	Thermal	Bhaya D *et al.*, 2007
HS Mushroom	20723	Thermal	Bhaya D *et al.*, 2007

REFERENCES

1. Tyson GW, Lo I, Baker BJ, Allen EE, Hugenholtz P, Banfield JF (2005) Genome-directed isolation of the key nitrogen fixer Leptospirillum ferrodiazotrophum sp. nov. from an acidophilic microbial community. *Appl Environ Microbiol.* 71(10):6319-24.
2. Dinsdale EA, Edwards RA et al. (2008). Functional metagenomic profiling of nine biomes. *Nature.* 452(7187):629-632.
3. Turnbaugh PJ, Ley RE, Mahowald MA, Magrini V, Mardis ER and Gordon JI (2006). An obesity-associated gut microbiome with increased capacity for energy harvest. *Nature* 444(7122):1027-1031.
4. Mou X, Sun S, Edwards RA, Hodson RE, Moran MA (2008) Bacterial carbon processing by generalist species in the coastal ocean. *Nature* 454(7179):708-711.
5. Bhaya D *et al.* (2007) Population level functional diversity in a microbial community revealed by comparative genomic and metagenomic analyses. *ISME J.*1(8):703-13.
