# Supplementary material for: HabiSign: a novel approach for comparison of metagenomes and rapid identification of habitat-specific sequences
Source: BMC Bioinformatics. 2011 Nov 30;12(Suppl 13):S9. doi: 10.1186/1471-2105-12-S13-S9 (PMC3278849; doi:10.1186/1471-2105-12-S13-S9)
Supplement: Additional File 2 — Computation and Comparison of Genealogical Indexes (GSIs) A pdf document describing the details of the computation and comparison of GSI values. These values are obtained for the various biome-level, species-level and phenotypic groups using the three methods (namely, HabiSign, the average tetranucleotide frequency approach and the dinucleotide relative abundance based approach) [file 1471-2105-12-S13-S9-S2.pdf]

COMPUTATION AND COMPARISON OF GENEALOGICAL SORTING INDEXES

GENEALOGICAL SORTING INDEXES

The concept of Genealogical Sorting Indexes (GSIs) was first introduced by Cummings *et al.* [1]. Given a set of observations belonging to different groups (represented in the form of a hierarchial tree), the Genealogical Sorting Index (GSI) value obtained for a particular group provides a quantitative measure of how closely the observations belonging to this group have clustered in the given tree. The GSI value ranges between the values of 0 and 1, where in a group obtains a GSI value of 1, if all the members of the group can be distinctly represented as a separate homogenous sub-clade of the tree (having no members belonging to any of the other groups). As the observations (belonging to a particular group) spread out, the GSI values for such groups fall below one.

The following figure illustrates these aspects.

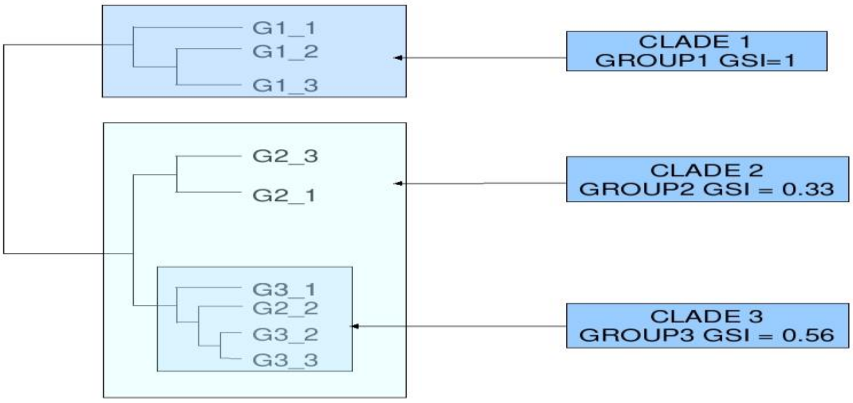

It is observed in the above figure, that samples belonging to group G1 have clustered distinctly as a separate sub-clade in the hierarchical tree (with no members of any other group as members of this sub-clade). The group G1 therefore obtains a GSI value of 1. In contrast, members belonging to groups G2 and G3 are observed to be spread out. It is observed that sample G2_2 belonging to group G2 has clustered closer to samples belonging to group G3. Furthermore, it is also observed that the sub-clade encompassing all three samples of group G3 is relatively smaller (thereby much more compact) as compared to the sub-clade encompassing all three samples belonging to group G2. In other words, the observations (i.e samples) belonging to group G3 have clustered together in a much more compact manner as compared to the observations belonging to group G2. Consequently the GSI value for group G3 (0.56) is observed to be higher than that obtained for group G2 (0.33).

Evaluating the clustering efficiency of Habisign, Average Tetranucleotide frequency-based approach, and the Dinucleotide Relative Abundance-based approach using Genealogical Sorting Indexes (GSIs):

Given that GSI values give a quantitative measure of the homogeneity of sub-clades obtained for observations belonging to different groups, the clustering efficiency of the three different methods (i.e. HabiSign, average tetranucleotide frequency based approach and the dinucleotide relative

abundance approach used by Willner *et al*.,) was evaluated using GSI values. The premise of this evaluation is as follows.

A method with a high clustering efficiency is expected to efficiently group/cluster metagenomic samples having similarities at biome, species or phenotypic levels. Consequently the tree (representing the clustering pattern) obtained with such a method is expected to contain sub-clades that are much more homogenous as compared to those obtained using the other methods. Consequently, the GSI values obtained using an efficient method are expected to be higher as compared to values obtained with other methods.

Based on the above premise, samples constituting the metagenomic data sets (used in the present study) were first categorized into groups. Each group contained samples having similarities at either biome-level or species-level or at the phenotypic-level. The metagenomic samples were then clustered using HabiSign, Average Tetranucleotide frequency-based approach, and the Dinucleotide Relative Abundance-based approach. The resulting clustering patterns (represented in the form of trees) obtained with all three methods were then evaluated by computing GSI values for the different groups. The details of the various datasets used in this analysis and the results obtained are given below.

DATASET 1: METAGENOMES SAMPLED FROM DIVERSE AQUATIC HABITATS

Grouping of Metagenomes

The diverse aquatic metagenomes were categorized into 8 biome-level groups as described below

METAGENOME	GROUP
Porites compressa 1	Coral
Porites compressa 2	Coral
Porites compressa 3	Coral
Porites compressa 4	Coral
Porites compressa 5	Coral
Porites compressa 6	Coral
Porites astreoides	Coral
Coral reef: Kingman	Coral Island
Coral reef: Palmyra	Coral Island
Coral reef: Tabuaeran	Coral Island
Coral reef: Christmas	Coral Island
Marine 1	Marine
Marine 2	Marine
Marine 3	Marine
Marine 4	Marine
Freshwater 1	Freshwater
Freshwater 2	Freshwater
Freshwater 3	Freshwater

Freshwater 4	Freshwater
Low salinity 1	Low Salinity
Low salinity 2	Low Salinity
Low salinity plasmids	Low Salinity
Medium salinity 1	Medium Salinity
Medium salinity 2	Medium Salinity
Medium salinity 3	Medium Salinity
Medium salinity 4	Medium Salinity
High salinity	High Salinity
HS Bison	Hot Spring
HS Octopus	Hot Spring
HS Mushroom	Hot Spring

Computation of GSI Values

The metagenomie signatures for the above metagenomes generated using each of the three methods (i.e., HabiSign, the average tetranucleotide approach as well as the dinucleotide relative abundance approach) were hierarchially clustered by providing the corresponding distance matrices as input to the Phylip package (using Neighbor joining algorithm with default parameters and no branch length). For computing the GSI values, the trees generated (shown in Figure 1A-C of the main manuscript) for each of the methods were subsequently provided as input to the Genealogical Sorting Index web-server (http://www.genealogicalsorting.org/). The GSI web-server takes in as input a rooted hierarchically tree showing the metagenomes as leaf nodes (in newick format) along with an assignment file that contains a mapping between each metagenome and its corresponding group (similar to Supplementary Table S1). The output generated from this server contains the GSI values for each individual group.

Comparison of GSI Values

The GSI values obtained for the different groups obtained using the three different methods are summarized below

GROUP	Genealogical Sorting Indexes (GSIs) Obtained Using		
	HabiSign	Average Tetranucleotide Frequency Approach	Dinucleotide relative abundance approach
Coral	0.68	0.49	0.49
Coral Island	0.72	0.44	0.27
Marine	1	1	1
Freshwater	0.72	0.3	0.72
Low Saline	0.23	0.14	0.14
Medium Saline	0.55	0.44	0.55
Hot Spring	0.47	1	1

Results in the above table indicate the following. Except for the group corresponding to the Hot-Spring biome, the GSI values obtained for the different groups using HabiSign is consistently equal to or higher than that obtained using the other two methods. This indicates that for the diverse aquatic metagenomes, the overall clustering efficiency obtained using the HabiSign signatures is higher as compared to those generated using either the average tetranucleotide approach or the dinucleotide relative abundance approach.

DATA SET 2: METAGENOMES FROM SIMILAR HABITATS BUT SAMPLED FROM DIFFERENT SPECIES

Grouping of Metagenomes

12 different gut metagenomes were categorized into four different groups based on the species from which they were sampled from. Details of the groups are given below

METAGENOMES	GROUP
Fish Morbid Gut	Fish
Fish Healthy Gut	Fish
Chicken Ceacum 1	Chicken
Chicken Ceacum 2	Chicken
Human Lean Gut	Human
Human Obese Gut	Human
Mouse Lean Gut	Mouse
Mouse Obese Gut	Mouse
Cow Rumen 1	Cow
Cow Rumen 2	Cow
Cow Rumen 3	Cow
Cow Rumen 4	Cow

Computation of GSI Values

In a manner similar to that described for the diverse aquatic metagenomes, the hierarchical trees generated for the gut metagenomes (shown in Figure 2A-C of the main manuscript) for each of the methods were provided as input (in the newick format) to the Genealogical Sorting Index web-server (http://www.genealogicalsorting.org/).

Comparison of GSI Values

The GSI values obtained for the different groups obtained using the three different methods are summarized below.

GROUP	Genealogical Sorting Indexes (GSIs) Obtained Using		
	**HabiSign**	**Average Tetranucleotide Frequency Approach**	**Dinucleotide relative abundance approach**
Fish	1	1	1
Chicken	1	1	1
Mouse	1	0.27	0.18
Human	1	0.12	0.45
Cow	0.25	0.45	0.21

It is observed that, using HabiSign, GSI values of 1 are obtained for all species level groups (except Cow). The lower GSI value obtained for the Cow group is due to the Cow Rumen 4 sample which is placed as an outlier as compared to the other Cow Rumen samples. This trend is in fact observed, for all the three methods (Figure 2A-C in the main manuscript). The probable reasons for this anomaly are already indicated in our taxonomic and specific-sequence analysis (in the main manuscript).

On the other hand, although the average tetranucleotide approach and the dinucleotide relative abundance approach are able to achieve a maximum GSI values of 1 for the Fish and Chicken group, the GSI values obtained for groups corresponding to Mouse and Human are seen to be noticeably lower than that obtained using HabiSign. This reaffirms the high clustering efficiency of HabiSign as compared to the other two methods.

DATA SET 3: METAGENOMES IDENTICAL AT HABITAT AND SPECIES LEVEL BUT EXHIBITING DIFFERENCES AT PHENOTYPE LEVEL

Grouping of Metagenomes

The three lean mouse gut metagenomes (Lean1, Lean2 and Lean3) were categorized as belonging to the 'Lean' group and the two obese metagenomes (Obese1 and Obese2) were classified as belonging to the 'Obese' group.

Computation of GSI values

The GSI values were computed by providing the hierarchical trees (Figure 3A-C) as input to the GSI computation web-server (http://www.genealogicalsorting.org/). The obtained GSI values for the Lean and Obese groups for three methods are shown below

Comparison of GSI Values

GROUP	Genealogical Sorting Indexes (GSIs) Obtained Using		
	**HabiSign**	**Average Tetranucleotide Frequency Approach**	**Dinucleotide relative abundance approach**
Lean	1	0.33	0.33
Obese	0.33	0.11	0.11

It is observed that the GSI values obtained using HabiSign for the Lean and Obese groups (1 and 0.33 respectively) were higher than that obtained using both the Average tetranucleotide frequency approach and the dinucleotide relative abundance based approach (0.33 and 0.11 respectively). This reaffirms the high clustering efficiency of HabiSign as compared to the other two methods.

REFERENCE

1. Cummings MP, Neel MC, Shaw KL. A genealogical approach to quantifying lineage divergence. *Evolution.* 2008 Sep;62(9):2411-22.
