# Supplementary material for: HabiSign: a novel approach for comparison of metagenomes and rapid identification of habitat-specific sequences
Source: BMC Bioinformatics. 2011 Nov 30;12(Suppl 13):S9. doi: 10.1186/1471-2105-12-S13-S9 (PMC3278849; doi:10.1186/1471-2105-12-S13-S9)
Supplement: Additional file 3 — Distribution of taxonomic assignments for the coral reef associated metagenomes A pdf document containing the distribution of taxonomic assignments (cumulated at phylum level) obtained using SPHINX for the coral reef associated metagenomes. [file 1471-2105-12-S13-S9-S3.pdf]

Supplementary Table 1: Distribution of taxonomic assignments* for the coral reef associated metagenomes

Archaeal Phyla	Percentage of sequences* assigned to Archaeal phyla										
	PA	PC1	PC2	PC3	PC4	PC5	PC6	Christmas	Palmyra	Tabuaeran	Kingman
Euryarchaeota	7.8	2.5	2.2	3.1	2.3	3.7	1.6	6.5	2.7	1.1	4.1
Crenarchaeota	6.2	3.2	3.8	2.5	3.8	0.0	2.7	4.7	4.6	6.7	4.8
Korarchaeota	0.4	0.0	0.0	0.0	0.0	0.0	0.0	0.0	0.2	0.0	0.0

Bacterial Phyla	Percentage of sequences* assigned to Bacterial Phyla										
	PA	PC1	PC2	PC3	PC4	PC5	PC6	Christmas	Palmyra	Tabuaeran	Kingman
Tenericutes	4.5	5.7	5.1	7.6	6.0	6.2	7.3	3.1	6.3	1.9	2.5
Fibrobacteres	0.1	0.0	0.0	0.0	0.0	0.0	0.0	0.0	0.0	0.0	0.0
Synergistetes	0.6	0.0	0.0	0.0	0.0	0.0	0.0	1.5	1.3	0.3	0.3
Chlorobi	0.7	0.0	0.0	0.0	0.0	0.0	0.0	0.8	0.5	0.0	0.5
Spirochaetes	3.0	5.6	9.3	5.5	7.1	4.4	5.9	4.5	3.9	1.7	4.1
Firmicutes	21.4	26.1	32.2	25.9	30.8	25.7	30.0	22.5	24.5	14.5	18.2
Verrucomicrobia	0.0	0.0	0.0	0.0	0.0	0.0	0.0	0.0	0.3	0.0	0.0
Aquificae	2.4	2.7	3.1	2.4	3.4	1.9	2.6	2.4	1.7	0.7	1.2
Thermotogae	2.9	0.9	1.4	0.0	1.2	0.0	0.9	3.5	1.0	0.3	1.4
Proteobacteria	35.3	19.1	23.1	19.9	25.6	25.3	24.4	28.9	32.6	40.0	37.1
Cyanobacteria	3.3	25.0	15.8	25.3	14.9	19.9	19.3	3.2	10.0	22.0	11.7
Actinobacteria	5.1	6.9	3.8	6.7	2.7	9.1	4.2	10.1	4.4	2.0	7.9
Planctomycetes	1.7	0.0	0.0	0.0	0.0	0.0	0.0	2.0	0.2	0.0	1.3
Chloroflexi	1.6	1.1	1.1	1.2	1.1	0.0	1.0	1.2	2.9	6.5	4.0
Bacteroidetes	1.8	0.0	0.0	0.0	0.0	0.0	0.0	0.8	0.8	0.9	1.2
Deinococcus-Thermus	1.0	0.9	0.0	0.0	1.2	3.8	0.0	4.4	2.1	1.4	0.0
Chlamydiae	0.2	0.0	0.0	0.0	0.0	0.0	0.0	0.0	0.0	0.0	0.0

* All assignments at or below phylum level were first cumulated to phylum level. Percentages shown in the above table are with respect to the sequences assigned at or below the level of phylum.

METAGENOME	DETAIL
PA	*Porites astreoides*
PC1	*Porites compressa 1*
PC2	*Porites compressa 2*
PC3	*Porites compressa 3*
PC4	*Porites compressa 4*
PC5	*Porites compressa 5*
PC6	*Porites compressa 6*
Christmas	Coral reef: Christmas
Palmyra	Coral reef: Palmyra
Tabuaeran	Coral reef: Tabuaeran
Kingman	Coral reef: Kingman
