# Supplementary material for: HabiSign: a novel approach for comparison of metagenomes and rapid identification of habitat-specific sequences
Source: BMC Bioinformatics. 2011 Nov 30;12(Suppl 13):S9. doi: 10.1186/1471-2105-12-S13-S9 (PMC3278849; doi:10.1186/1471-2105-12-S13-S9)
Supplement: Additional file 4 — Taxonomic analysis of sequences identified as specific to the Coral PA and the Coral PC metagenomes A pdf document containing the distribution of taxonomic assignments (cumulated at phylum level) obtained using SPHINX for the sequences identified as specific to the Coral PA and Coral PC (Coral PC 1-6) metagenomes. [file 1471-2105-12-S13-S9-S4.pdf]

Supplementary Table: Comparison of taxonomic assignments obtained for the sequences as specific to the PA and PC metagenomes (PC1-6)

Phylum Name	% Sequences Assigned		Relative Ratio (X/Y)	Inference
	PA Metagenome specific (X)	PC Metagenomes specific (Y)		
Euryarchaeota	10.5	0.0	274.8	High in PA
Proteobacteria	55.6	27.3	2.0	High in PA
Thermotogae	6.4	0.0	N.A	Only in PA
Planctomycetes	10.0	0.0	N.A	Only in PA
Firmicutes	4.6	9.4	0.5	High in PC
Actinobacteria	5.0	13.2	0.4	High in PC
Cyanobacteria	1.9	40.5	0.0	High in PC
Deinococcus-Thermus	0.0	7.6	0.0	Only in PC
