# Supplementary material for: HabiSign: a novel approach for comparison of metagenomes and rapid identification of habitat-specific sequences
Source: BMC Bioinformatics. 2011 Nov 30;12(Suppl 13):S9. doi: 10.1186/1471-2105-12-S13-S9 (PMC3278849; doi:10.1186/1471-2105-12-S13-S9)
Supplement: Additional file 5 — Distribution of taxonomic assignments for metagenomes obtained from habitats of varying salinity A pdf document containing the distribution of taxonomic assignments (cumulated at the phylum level) obtained using SPHINX for the metagenomes sampled from habitats of varying salinity. [file 1471-2105-12-S13-S9-S5.pdf]

Supplementary Table 1: Distribution of taxonomic assignments* for metagenomes obtained from habitats of varying salinity

Archaeal Phyla	Percentage of sequences* assigned											
	FW-1	FW-2	FW-3	FW-4	LS-Plas	LS-2	LS-1	MS-1	MS-2	MS-3	MS-4	HS
Euryarchaeota	1.8	0.0	0.0	2.1	2.9	2.7	4.03	0.0	4.6	3.4	3.6	29.2
Crenarchaeota	0.0	2.2	0.0	1.4	1.8	3.2	0.92	0.0	0.0	0.0	0.0	0.0

Bacterial Phyla	Percentage of sequences* assigned											
	FW-1	FW-2	FW-3	FW-4	LS-Plas	LS-2	LS-1	MS-1	MS-2	MS-3	MS-4	HS
Tenericutes	4.9	6.6	6.2	6.0	15.9	18.9	1.46	0	0.42	0	1.67	0
Bacteroidetes	1.9	1.6	1.3	1.9	0.0	0.0	0.59	0	0.39	0	0	0
Spirochaetes	4.9	6.7	3.3	5.0	3.7	2.9	1.9	0	0.81	0	1.5	0
Firmicutes	25.9	26.3	12.9	24.8	23.0	26.9	8.55	0	3.32	4.56	9.51	2.4
Proteobacteria	52.7	44.8	55.7	44.5	35.1	33.9	48.31	49.32	26.88	26.93	44.63	20.58
Actinobacteria	7.9	7.0	16.9	6.0	10.4	8.5	26.98	50.68	56.57	65.11	36.95	47.06
Chlorobi	0.0	0.8	0.6	0.7	0.3	0.0	0.39	0	0	0	0	0
Planctomycetes	0.0	1.4	0.7	1.2	1.7	0.0	2.22	0	0.73	0	1.32	0
Cyanobacteria	0.0	2.6	1.2	2.0	1.3	3.1	1.63	0	0.68	0	0	0
Chloroflexi	0.0	0.0	1.2	1.3	1.0	0.0	1.31	0	0.8	0	0	0
Synergistetes	0.0	0.0	0.0	0.2	0.0	0.0	0.21	0	0	0	0	0
Fusobacteria	0.0	0.0	0.0	0.6	0.0	0.0	0.0	0.0	0.0	0.0	0.0	0.0
Aquificae	0.0	0.0	0.0	1.4	1.6	0.0	0.67	0	0.37	0	0.84	0
Thermotogae	0.0	0.0	0.0	0.5	0.7	0.0	0.59	0	0	0	0	0.77
Deinococcus-Thermus	0.0	0.0	0.0	0.4	0.6	0.0	0.23	0	0.33	0	0	0
Verrumicrobia	0.0	0.0	0.0	0.0	0.0	0.0	0	0	4.13	0	0	0

* All assignments at or below phylum level were first cumulated to phylum level. Percentages shown in the above table are with respect to the sequences assigned at or below the level of phylum.

METAGENOME	DETAIL
**FW-1**	Freshwater 1
**FW-2**	Freshwater 2
**FW-3**	Freshwater 3
**FW-4**	Freshwater 4
**LS-Plas**	Low salinity plasmids
**LS-2**	Low salinity 2
**LS-1**	Low salinity 1
**MS-1**	Medium salinity 1
**MS-2**	Medium salinity 2
**MS-3**	Medium salinity 3
**MS-4**	Medium salinity 4
**HS**	High salinity
