# Supplementary material for: HabiSign: a novel approach for comparison of metagenomes and rapid identification of habitat-specific sequences
Source: BMC Bioinformatics. 2011 Nov 30;12(Suppl 13):S9. doi: 10.1186/1471-2105-12-S13-S9 (PMC3278849; doi:10.1186/1471-2105-12-S13-S9)
Supplement: Additional file 6 — Distribution of taxonomic assignments for the cow rumen metagenomes A pdf document containing the distribution of taxonomic assignments (to various phyla) obtained using the SPHINX algorithm for the cow rumen metagenomes. [file 1471-2105-12-S13-S9-S6.pdf]

Supplementary Table: Distribution of taxonomic assignments* for the cow rumen metagenomes

**Metagenome**	**Percentage of sequences* assigned**					
	**Firmicutes**	**Proteobacteria**	**Bacteroidetes**	**Actinobacteria**	**Euryarchaeota**	**Cyanobacteria**
Cow Rumen 1	27.21	50.26	6.59	11.24	3.27	1.43
Cow Rumen 2	27.70	47.14	9.82	10.65	3.46	1.22
Cow Rumen 3	26.20	47.36	6.48	12.80	5.69	1.47
Cow Rumen 4	16.16	76.17	2.05	2.85	1.23	1.54

* All assignments at or below phylum level were first cumulated to phylum level. Percentages shown in the above table are with respect to the sequences assigned at or below the level of phylum.
