# Supplementary material for: HabiSign: a novel approach for comparison of metagenomes and rapid identification of habitat-specific sequences
Source: BMC Bioinformatics. 2011 Nov 30;12(Suppl 13):S9. doi: 10.1186/1471-2105-12-S13-S9 (PMC3278849; doi:10.1186/1471-2105-12-S13-S9)
Supplement: Additional file 7 — Taxonomic analysis of sequences identified as specific to the Cow rumen 4 and Cow Rumen 1-3 metagenomes A pdf document containing the distribution of taxonomic assignments (cumulated at phylum level) obtained using SPHINX for the sequences identified as specific to the Cow Rumen 4 and the other Cow Rumen (Cow Rumen 1-3) metagenomes. [file 1471-2105-12-S13-S9-S7.pdf]

.

Supplementary Table: Comparison of taxonomic assignments obtained for the sequences as specific to the Cow Rumen 4 sample and those identified as specific to Cow Rumen samples 1-3

Phylum Name	% Sequences Assigned		Relative Ratio (X/Y)	Inference
	Cow Rumen 4 specific (X)	Cow Rumen 1-3 Specific (Y)		
Proteobacteria	84.4	59.9	1.4	High in Cow Rumen 4
Firmicutes	9.9	6.0	1.7	High in Cow Rumen 4
Euryarchaeota	0.0	6.3	N.A	Only in Cow Rumen 1-3
Actinobacteria	0.0	17.5	N.A	Only in Cow Rumen 1-3
Bacteroidetes	0.0	3.3	N.A	Only in Cow Rumen 1-3
