# Supplementary material for: HabiSign: a novel approach for comparison of metagenomes and rapid identification of habitat-specific sequences
Source: BMC Bioinformatics. 2011 Nov 30;12(Suppl 13):S9. doi: 10.1186/1471-2105-12-S13-S9 (PMC3278849; doi:10.1186/1471-2105-12-S13-S9)
Supplement: Additional file 8 — Distribution of taxonomic assignments from lean and obese mouse gut metagenomes. A pdf document containing the distribution of taxonomic assignments (to various phyla) obtained using the SPHINX algorithm for the lean and obese mouse gut metagenomes. [file 1471-2105-12-S13-S9-S8.pdf]

Supplementary Table: Distribution of taxonomic assignments* from lean and obese mouse gut metagenomes.

Phylum Name	Lean1	Lean 2	Lean 3	Obese 1	Obese 2
Euryarchaeota	3.13	4.09	4.65	7.54	4.48
Proteobacteria	36.57	35.58	39.65	40.29	42.09
Actinobacteria	3.28	2.76	3.25	0.00	3.50
Bacteroidetes	50.55	49.23	38.07	34.44	36.41
Cyanobacteria	0.00	2.57	3.54	3.74	2.82
Firmicutes	6.47	5.77	10.83	13.98	10.70
Bacteroidetes/Firmicutes ratio	7.81	8.53	3.51	2.46	3.40

* All assignments at or below phylum level were first cumulated to phylum level. Percentages shown in the above table are with respect to the sequences assigned at or below the level of phylum.
