# Supplementary material for: HabiSign: a novel approach for comparison of metagenomes and rapid identification of habitat-specific sequences
Source: BMC Bioinformatics. 2011 Nov 30;12(Suppl 13):S9. doi: 10.1186/1471-2105-12-S13-S9 (PMC3278849; doi:10.1186/1471-2105-12-S13-S9)
Supplement: Additional file 9 — Genealogical Sorting Index (GSI) based comparative analysis of clustering efficiencies using simulated metagenomic data sets. A pdf document describing a GSI based comparative analysis of the clustering efficiencies of HabiSign, the average tetranucleotide frequency approach and the dinucleotide relative abundance based approach on simulated metagenomic data sets [file 1471-2105-12-S13-S9-S9.pdf]

EVALUATING THE CLUSTERING EFFICIENCIES OF THE THREE METHODS ON SIMULATED METAGENOMIC DATA SETS USING GENEALOGICAL SORTING INDEXES (GSIs)

The clustering efficiencies of the three methods (i.e. HabiSign, average tetranucleotide frequency based approach, dinucleotide relative abundance approach) were evaluated on two simulated metagenomic data sets. The first set contained metagenomic groups having very distinct taxonomic composition. All three methods are expected to cluster these groups with equal efficiency. In contrast, the second simulated set contained metagenomic groups having similarity with respect to the constituent species. However, there were subtle differences in the relative abundance of the constituent species. A method with a high clustering efficiency is expected to capture these subtle differences and appropriate cluster the metagenomic samples belonging to different groups.

The details of the simulated metagenomic data sets, the methodology adopted for evaluation and the results obtained are summarized in this document.

SIMULATED DATA SET 1

This data set consisted of nine simulated metagenomic samples (M11, M12, M13, M21, M22, M23, M31, M32, M33). The taxonomic composition of these samples were simulated in such a manner that they could be classified into three different groups. The details of these groups are provided below. Note that the species constituting these three groups are entirely different.

(A) GROUP1: Consists of metagenomic samples M11, M12 and M13. The relative abundance of the constituent species (in terms of the number of sequences) are also indicated below.

SPECIES	No. of sequences in metagenome		
	M11	M12	M13
Aeromonas hydrophila	1436	6351	2101
Akkermansia muciniphila	5912	5182	2477
Bifidobacterium longum	2670	7323	5259
Buchnera aphidicola	6056	4457	2405
Desulfovibrio desulfuricans	7199	6053	1792
Dichelobacter nodosus	7555	2469	1546
Dictyoglomus thermophilum	1374	4548	4392
Gloeobacter violaceus	3098	224	4293
Gramella forsetii	5659	2492	7176
Halothermothrix orenii	1457	892	203
Herpetosiphon aurantiacus	7268	1973	1180
Legionella pneumophila	6219	7916	411
Marinomonas sp. MWYL1	1421	6708	3167
Methanospirillum hungatei	3206	5048	918
Natranaerobius thermophilus	4933	791	4675
Natronomonas_pharaonis	2144	4449	1840
Nautilia profundicola	4163	4392	6049
Nitrosopumilus maritimus	4888	4076	3433
Prochlorococcus marinus	7052	7385	2732
Rhodospirillum centenum	1107	3037	3405
Roseiflexus sp. RS-1	6685	3169	4779
Rubrobacter xylanophilus	3437	2491	6572
Solibacter usitatus	5597	4103	3656
Sphingopyxis alaskensis	7524	7520	954
Thermoanaerobacter sp. X514	1117	116	7981
Thermodesulfovibrio yellowstonii	6283	529	4262
Thermoplasma volcanium	2390	3244	7991
Thiobacillus denitrificans	2352	5345	3473

B) GROUP 2: Consists of metagenomic samples M21, M22 and M23. The relative abundance of the constituent species (in terms of the number of sequences) are also indicated below.

SPECIES	No. of sequences in metagenome		
	M21	M22	M23
Acholeplasma laidlawii	6115	2567	3980
Acidithiobacillus ferrooxidans	7735	1758	480
Acidobacterium capsulatum	1627	3036	6384
Archaeoglobus fulgidus	5517	4818	7229
Aster yellows witches-broom phytoplasma	7558	7	736
Bacteroides fragilis	2659	4771	5854
Desulfotalea psychrophila	6397	4152	4129
Fusobacterium nucleatum	2222	3129	2847
Gemmatimonas aurantiaca	4564	7122	7439
Lawsonia intracellularis	6408	3868	4190
Mesoplasma florum	5267	5295	5149
Methanococcus maripaludis	5606	2361	3546
Methanopyrus kandleri	3542	3638	6308
Methanosarcina barkeri	7164	1934	6516
Methanosphaera stadtmanae	1437	7907	3140
Methylobacillus flagellatus	5184	21	1026
Methylococcus capsulatus	4090	5492	648
Nitrosococcus oceani	3193	4671	5277
Nitrosospira multiformis	3070	4043	2748
Pelobacter propionicus	5250	106	7675
Phenylobacterius zucineum	6513	3429	4690
Pyrococcus abyssi	4860	6136	6838
Salinibacter ruber	2284	6574	7787
Silicibacter sp. TM1040	4689	6056	7654
Sorangium cellulosum	1932	1433	4073
Staphylothermus marinus	5634	3503	3787
Sulfolobus tokodaii	7462	3942	1326
Syntrophus aciditrophicus	3658	6826	6158
Thermofilum pendens	7550	5577	6554
Thermomicrobium roseum	391	3387	2354
Thermus thermophilus	7367	1984	5157
Vibrio cholerae	4647	7589	5372
Yersinia pseudotuberculosis	2738	6934	7595

(C) GROUP 3: Consists of metagenomic samples M31, M32 and M33. The relative abundance of the constituent species (in terms of the number of sequences) are also indicated below.

SPECIES	No. of sequences in metagenome		
	M31	M32	M33
Acidovorax avenae	4191	6497	5482
Agrobacterium tumefaciens	2433	7035	4643
Aquifex aeolicus	5217	4209	6380
Bacillus halodurans	1	1600	3018
Bdellovibrio bacteriovorus	4622	6298	6021
Bordetella pertussis	7381	5296	751
Burkhoderia cenocephacia	3985	2338	2179
Campylobacter jejuni	5322	2700	5715
Chlamydophila pneumoniae	1760	7212	4724
Chlorobium chlorochromatii	3952	1653	3657
Clostridium tetani	4772	6032	7837
Corynebacterium diphtheriae	6837	1084	2626
Escherichia coli	716	7475	5299
Francisella philomiragia	6171	7739	561
Haemophilus influenzae	2932	3484	2325
Helicobacter pylori	5473	1413	7003
Magnetococcus sp. MC-1	2013	2456	4254
Mycobacterium tuberculosis	3199	5722	7795
Mycoplasma pneumoniae	4953	2008	3294
Neisseria meningitidis	4884	2997	3028
Nostoc spp. 7120	4133	956	6205
Pseudomonas aeruginosa	3765	6623	4209
Rhodopseudomonas palustris	5738	4250	4312
Rickettsia bellii	3920	7592	385
Salmonella enterica	5154	6456	1790
Staphylococcus aureus	66	4501	949
Streptococcus pyogenes	7688	3077	4681
Thermotoga maritima	2460	7956	5354
Wigglesworthia brevipalpis	2054	7916	3290
Xanthomonas campestris	1485	6806	4444

COMPUTATION AND COMPARISON OF GSI VALUES FOR SIMULATED DATA SET 1

The nine simulated metagenomic samples (belong to the above three groups) were clustered using HabiSign, the average tetranucleotide frequency approach, and the dinucleotide relative abundance based approach. Hierarchical trees were obtained by providing the individual distance matrices (generated using the three methods) as inputs to the neighbor joining algorithm (with default parameters) of the Phylip package. The GSIs for the three different groups (obtained using the three methods) were computed by providing the hierarchical trees generated for each of the individual methods as inputs to the online GSI computation web-server available at http://www.genealogicalsorting.org/ . A comparison of GSI values obtained for the three different

groups using the three methods is given below

GROUPs	GSI Values obtained using		
	**HabiSign**	**Average Tetranucleotide frequencies**	**Dinucleotide relative abundances**
GROUP1	1	1	1
GROUP2	1	1	1
GROUP3	1	1	1

ANALYSIS

As expected, it is observed that all the three methods obtained a maximum GSI value for the three different groups. These results indicate that all the three methods perform equally when the different groups have entirely different taxonomic composition.

SIMULATED DATA SET 2:

This simulated set contains metagenomic groups having similarity with respect to the constituent species. However, across groups, there are subtle differences with respect to the relative abundance of these species. A method with a high clustering efficiency is expected to capture these subtle differences and appropriate cluster the metagenomic samples belonging to different groups.

Group 1 (consisting of the simulated samples G11, G12 and G13) had all the constituent species present in roughly equal proportions. On the other hand, the Groups 2 and 3 displayed the classic 'long tail' effect wherein the first 9 species were represented in much higher amounts (number of reads > 4000) as compared to the other species (number of reads in the range 200 – 400). This pattern of taxonomic composition is generally observed in real metagenomic data sets.

Furthermore, the metagenomes constituting groups 2 and 3 displayed subtle variations in their taxonomic composition. Metagenomes belonging to Group 2 had a relatively lower abundance of species *Francisella philomiragia*, *Wigglesworthia brevipalpis* and *Staphylococcus aureus* (number of reads ~ 200) as compared to Group 3. On the other hand, metagenomes belonging to Group 3 had a relatively lower abundance of species *Rickettsia bellii, Campylobacter jejuni, Haemophilus influenzae and Clostridium tetani* (number of reads ~ 200) as compared to Group 2.

The details of the three groups constituting this simulated metagenomic data set is given below.

(A) GROUP 1: Consists of metagenomic samples G11, G12 and G13. The relative abundance of the constituent species (in terms of the number of sequences) are also indicated below.

SPECIES	No. of sequences in metagenome		
	G11	G12	G13
Acidovorax avenae	1135	1183	1050
Bordetella pertussis	1104	1138	1066
Pseudomonas aeruginosa	1067	1036	1179
Mycobacterium tuberculosis	1165	1073	1065
Xanthomonas campestris	1051	1022	1055
Rhodopseudomonas palustris	1189	1084	1093
Magnetococcus sp. MC-1	1091	1159	1087
Burkhoderia cenocephacia	1126	1111	1190
Agrobacterium tumefaciens	1053	1092	1123
Corynebacterium diphtheriae	1091	1177	1065
Francisella philomiragia	1015	1103	1093
Staphylococcus aureus	1078	1151	1064
Bdellovibrio bacteriovorus	1198	1066	1011
Chlamydophila pneumoniae	1190	1056	1163
Aquifex aeolicus	1024	1024	1144
Escherichia coli	1028	1124	1104
Thermotoga maritima	1112	1108	1175
Nostoc spp. 7120	1094	1093	1022
Bacillus halodurans	1021	1163	1012
Streptococcus pyogenes	1164	1190	1097
Helicobacter pylori	1126	1143	1023
Rickettsia bellii	1185	1188	1092
Chlorobium chlorochromatii	1184	1167	1178
Wigglesworthia brevipalpis	1035	1124	1006
Salmonella enterica	1000	1179	1137
Mycoplasma pneumoniae	1094	1118	1071
Campylobacter jejuni	1093	1170	1074
Haemophilus influenzae	1044	1112	1078
Clostridium tetani	1044	1179	1058
Neisseria meningitidis	1114	1101	1077

(B) GROUP 2: Consists of metagenomic samples G21, G22 and G23. The relative abundance of the constituent species (in terms of the number of sequences) are also indicated below.

SPECIES	No. of sequences in metagenome		
	G21	G22	G23
Acidovorax avenae	4084	4152	4112
Bordetella pertussis	4008	4031	4144
Pseudomonas aeruginosa	4073	4002	4133
Mycobacterium tuberculosis	4109	4086	4083
Xanthomonas campestris	4164	4096	4021
Rhodopseudomonas palustris	4156	4009	4142
Magnetococcus sp. MC-1	4107	4198	4030
Burkhoderia cenocephacia	4037	4065	4080
Agrobacterium tumefaciens	4187	4060	4135
Corynebacterium diphtheriae	403	408	407
Francisella philomiragia	***273***	***227***	***232***
Staphylococcus aureus	***220***	***232***	***275***
Bdellovibrio bacteriovorus	459	418	491
Chlamydophila pneumoniae	449	435	465
Aquifex aeolicus	456	490	447
Escherichia coli	474	466	401
Thermotoga maritima	410	464	405
Nostoc spp. 7120	466	499	412
Bacillus halodurans	463	442	400
Streptococcus pyogenes	422	400	498
Helicobacter pylori	454	475	457
Rickettsia bellii	494	471	474
Chlorobium chlorochromatii	444	434	423
Wigglesworthia brevipalpis	***222***	***234***	***283***
Salmonella enterica	422	401	414
Mycoplasma pneumoniae	463	444	422
Campylobacter jejuni	418	440	405
Haemophilus influenzae	421	407	450
Clostridium tetani	464	496	485
Neisseria meningitidis	402	405	464

(C) GROUP 3: Consists of metagenomic samples G31, G32 and G33. The relative abundance of the constituent species (in terms of the number of sequences) are also indicated below.

SPECIES	No. of sequences in metagenome		
	G21	G22	G23
Acidovorax avenae	4059	4153	4193
Bordetella pertussis	4177	4122	4003
Pseudomonas aeruginosa	4051	4162	4194
Mycobacterium tuberculosis	4138	4138	4049
Xanthomonas campestris	4172	4140	4015
Rhodopseudomonas palustris	4041	4036	4088
Magnetococcus sp. MC-1	4033	4026	4159
Burkhoderia cenocephacia	4045	4048	4059
Agrobacterium tumefaciens	4190	4066	4055
Corynebacterium diphtheriae	482	412	401
Francisella philomiragia	496	434	406
Staphylococcus aureus	440	452	419
Bdellovibrio bacteriovorus	495	493	480
Chlamydophila pneumoniae	422	458	426
Aquifex aeolicus	498	488	414
Escherichia coli	487	456	466
Thermotoga maritima	437	415	420
Nostoc spp. 7120	419	498	401
Bacillus halodurans	439	412	494
Streptococcus pyogenes	451	449	456
Helicobacter pylori	420	422	468
Rickettsia bellii	***230***	***270***	***202***
Chlorobium chlorochromatii	458	498	493
Wigglesworthia brevipalpis	473	423	493
Salmonella enterica	466	485	456
Mycoplasma pneumoniae	457	415	424
Campylobacter jejuni	***281***	***269***	***256***
Haemophilus influenzae	***235***	***294***	***272***
Clostridium tetani	***206***	***241***	***223***
Neisseria meningitidis	415	477	414

COMPUTATION AND COMPARISON OF GSI VALUES FOR SIMULATED DATA SET 2

The nine simulated metagenomic samples (belong to the above three groups) were clustered using HabiSign, the average tetranucleotide frequency approach, and the dinucleotide relative abundance based approach. Hierarchical trees were obtained by providing the individual distance matrices (generated using the three methods) as inputs to the neighbor joining algorithm (with default parameters) of the Phylip package. The GSIs for the three different groups (obtained using the three methods) were computed by providing the hierarchical trees generated for each of the individual methods as inputs to the online GSI computation web-server available at http://www.genealogicalsorting.org/ . A comparison of GSI values obtained for the three different groups using the three methods is given below

GROUPs	GSI Values obtained using		
	**HabiSign**	**Average Tetranucleotide frequencies**	**Dinucleotide relative abundances**
GROUP1	1	1	1
GROUP2	1	0.56	1
GROUP3	1	0.33	0.4

It was observed that the three different methods attained a GSI value of 1 for Group 1. Moreover, HabiSign is observed to have GSI values of 1 for group 2 and 3 as well. These results indicate that the HabiSign approach is able to efficiently identify the subtle variations in the composition of metagenomes.

In contrast, using the average tetranucleotide frequencies based approach is observed to result in lower GSI values (0.56 and 0.33 respectively) for groups 2 and 3. Similarly, although the dinucleotide relative abundance based approach was able to attain a GSI value of 1 for Group 2, the GSI value obtained for Group 3 was observed to be 0.4.
